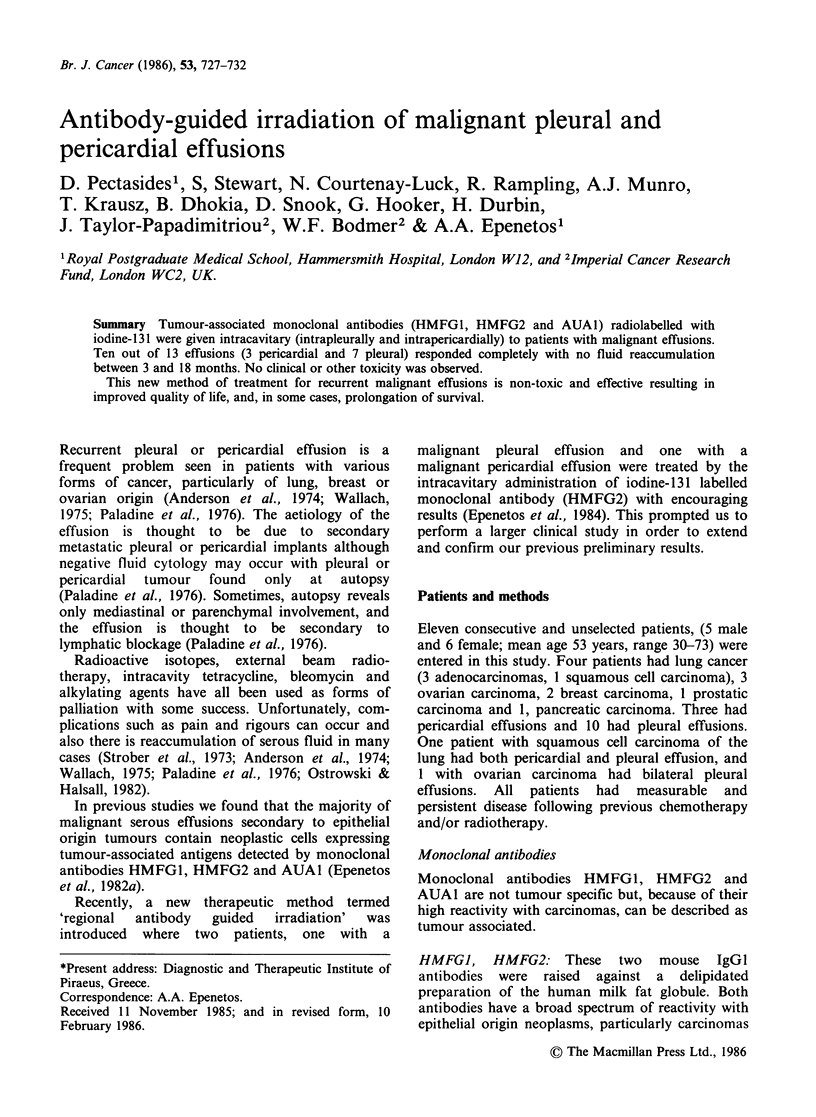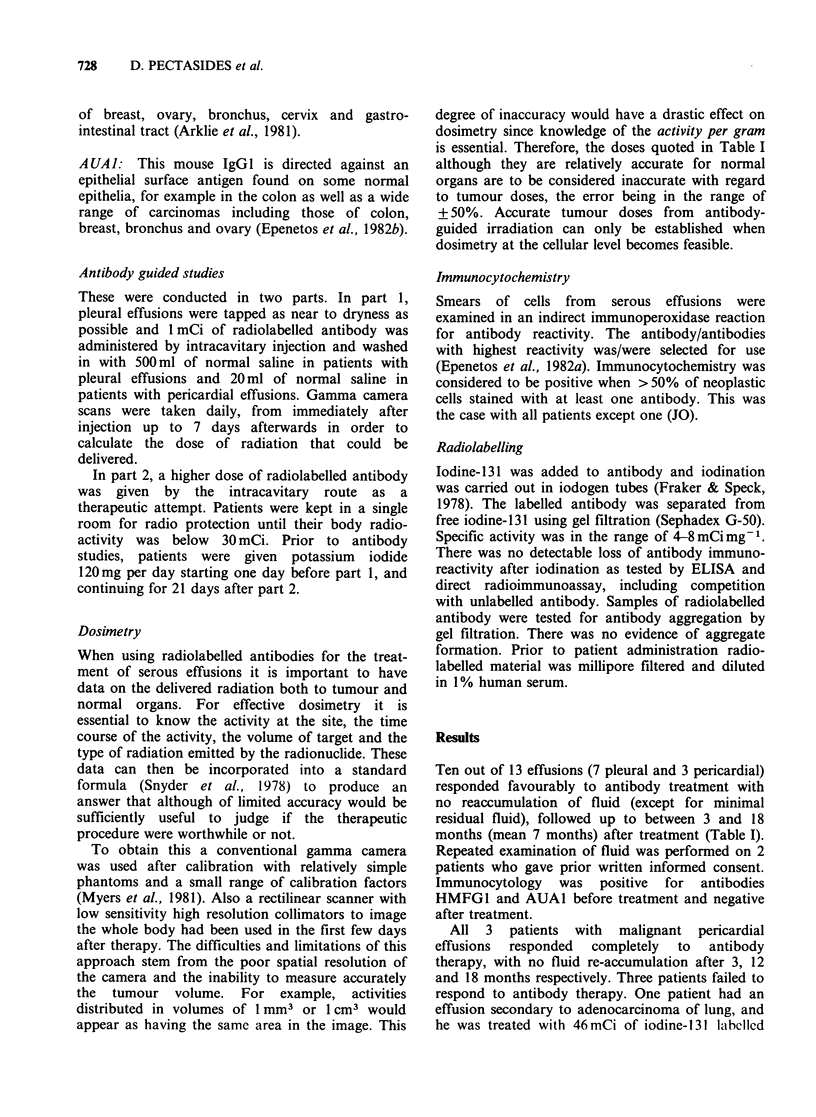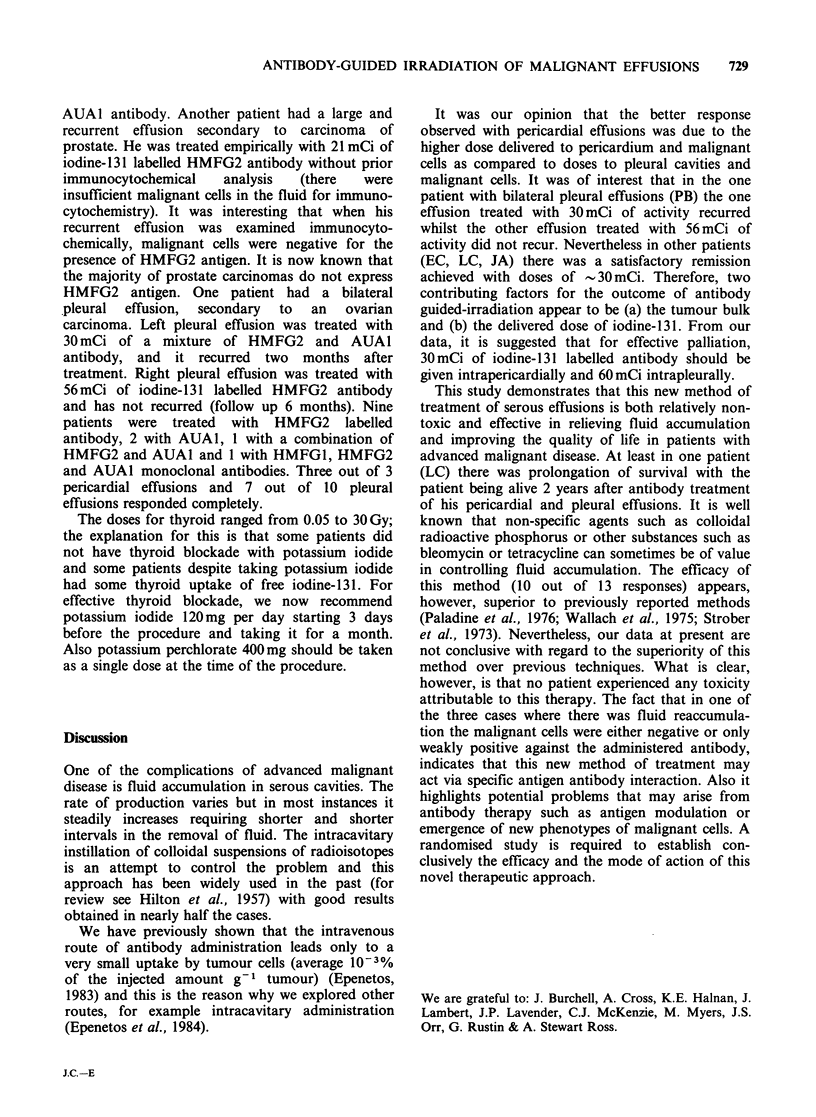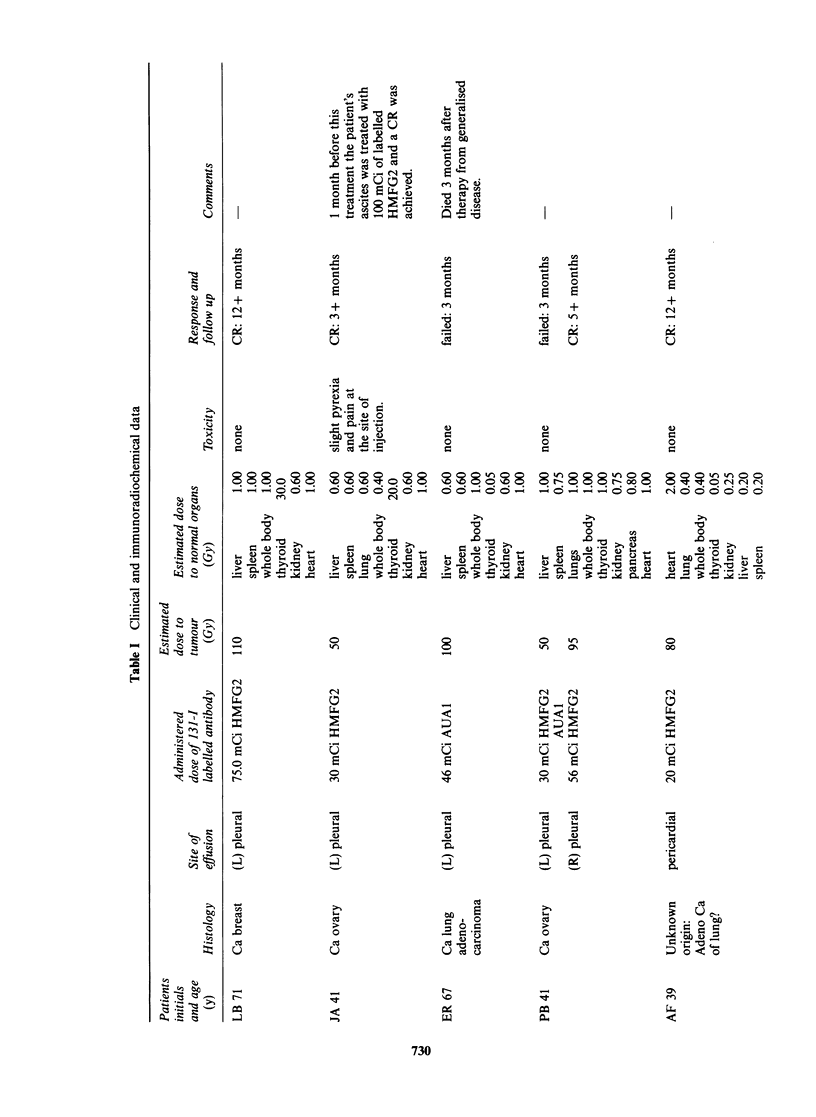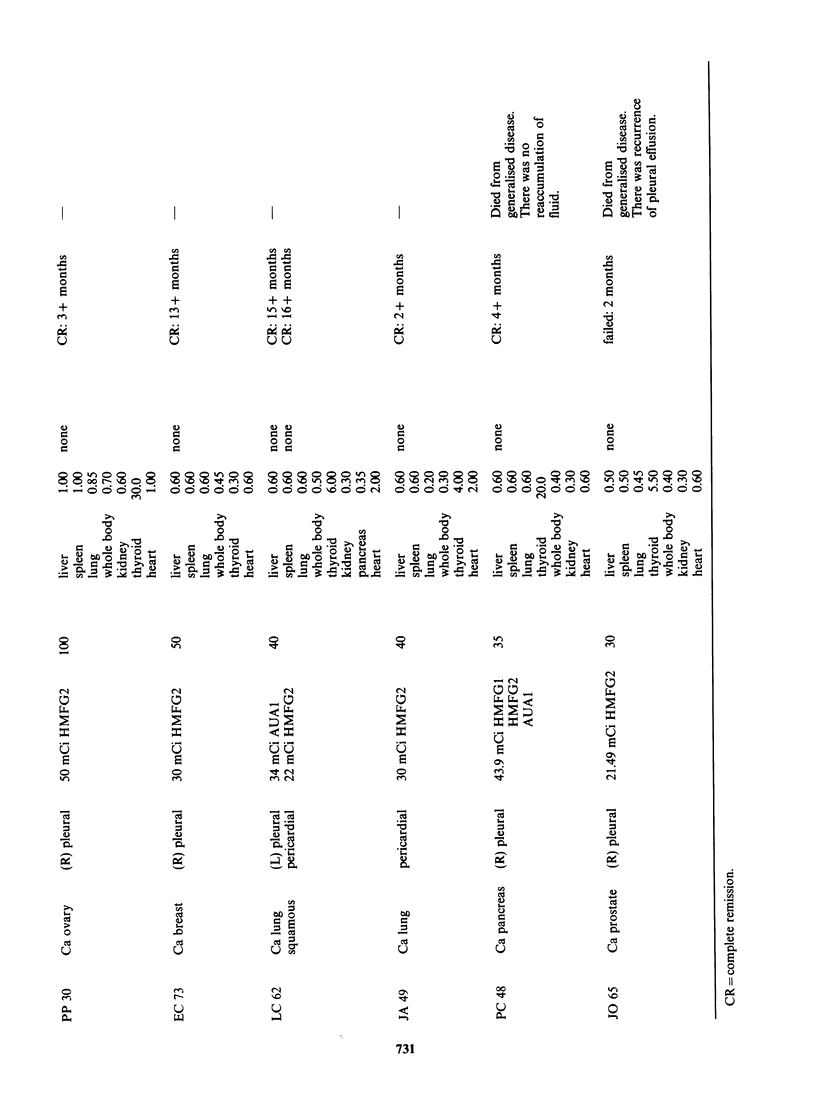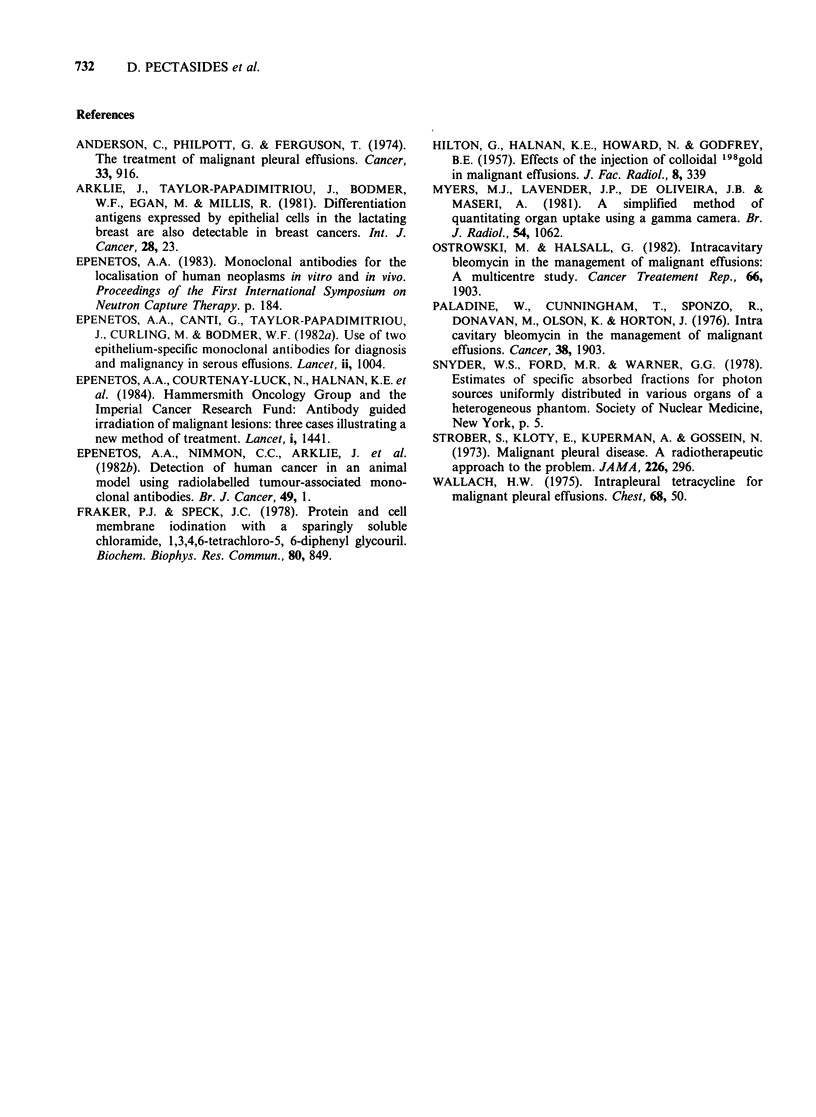# Antibody-guided irradiation of malignant pleural and pericardial effusions.

**DOI:** 10.1038/bjc.1986.125

**Published:** 1986-06

**Authors:** D. Pectasides, S. Stewart, N. Courtenay-Luck, R. Rampling, A. J. Munro, T. Krausz, B. Dhokia, D. Snook, G. Hooker, H. Durbin

## Abstract

Tumour-associated monoclonal antibodies (HMFG1, HMFG2 and AUA1) radiolabelled with iodine-131 were given intracavitary (intrapleurally and intrapericardially) to patients with malignant effusions. Ten out of 13 effusions (3 pericardial and 7 pleural) responded completely with no fluid reaccumulation between 3 and 18 months. No clinical or other toxicity was observed. This new method of treatment for recurrent malignant effusions is non-toxic and effective resulting in improved quality of life, and, in some cases, prolongation of survival.


*Br. J. Cancer* (1986), **53**, 727–732

### Antibody-guided irradiation of malignant pleural and pericardial effusions

D. Pectasides[1], S, Stewart, N. Courtenay-Luck, R. Rampling, A.J. Munro, T. Krausz, B. Dhokia, D. Snook, G. Hooker, H. Durbin, J. Taylor-Papadimitriou[2], W.F. Bodmer[2] & A.A. Epenetos[1]

[1]*Royal Postgraduate Medical School, Hammersmith Hospital, London W12, and* [2]*Imperial Cancer Research Fund, London WC2, UK.*

**Summary** Tumour-associated monoclonal antibodies (HMFG1, HMFG2 and AUA1) radiolabelled with iodine-131 were given intracavitary (intrapleurally and intrapericardially) to patients with malignant effusions. Ten out of 13 effusions (3 pericardial and 7 pleural) responded completely with no fluid reaccumulation between 3 and 18 months. No clinical or other toxicity was observed.

This new method of treatment for recurrent malignant effusions is non-toxic and effective resulting in improved quality of life, and, in some cases, prolongation of survival.

Recurrent pleural or pericardial effusion is a frequent problem seen in patients with various forms of cancer, particularly of lung, breast or ovarian origin (Anderson *et al.*, 1974; Wallach, 1975; Paladine *et al.*, 1976). The aetiology of the effusion is thought to be due to secondary metastatic pleural or pericardial implants although negative fluid cytology may occur with pleural or pericardial tumour found only at autopsy (Paladine *et al.*, 1976). Sometimes, autopsy reveals only mediastinal or parenchymal involvement, and the effusion is thought to be secondary to lymphatic blockage (Paladine *et al.*, 1976).

Radioactive isotopes, external beam radiotherapy, intracavity tetracycline, bleomycin and alkylating agents have all been used as forms of palliation with some success. Unfortunately, complications such as pain and rigours can occur and also there is reaccumulation of serous fluid in many cases (Strober *et al.*, 1973; Anderson *et al.*, 1974; Wallach, 1975; Paladine *et al.*, 1976; Ostrowski & Halsall, 1982).

In previous studies we found that the majority of malignant serous effusions secondary to epithelial origin tumours contain neoplastic cells expressing tumour-associated antigens detected by monoclonal antibodies HMFG1, HMFG2 and AUA1 (Epenetos *et al.*, 1982*a*).

Recently, a new therapeutic method termed 'regional antibody guided irradiation' was introduced where two patients, one with a malignant pleural effusion and one with a malignant pericardial effusion were treated by the intracavitary administration of iodine-131 labelled monoclonal antibody (HMFG2) with encouraging results (Epenetos *et al.*, 1984). This prompted us to perform a larger clinical study in order to extend and confirm our previous preliminary results.

*Present address: Diagnostic and Therapeutic Institute of Piraeus, Greece.

Correspondence: A.A. Epenetos.

Received 11 November 1985; and in revised form, 10 February 1986.

### Patients and methods

Eleven consecutive and unselected patients, (5 male and 6 female; mean age 53 years, range 30–73) were entered in this study. Four patients had lung cancer (3 adenocarcinomas, 1 squamous cell carcinoma), 3 ovarian carcinoma, 2 breast carcinoma, 1 prostatic carcinoma and 1, pancreatic carcinoma. Three had pericardial effusions and 10 had pleural effusions. One patient with squamous cell carcinoma of the lung had both pericardial and pleural effusion, and 1 with ovarian carcinoma had bilateral pleural effusions. All patients had measurable and persistent disease following previous chemotherapy and/or radiotherapy.

### *Monoclonal antibodies*

Monoclonal antibodies HMFG1, HMFG2 and AUA1 are not tumour specific but, because of their high reactivity with carcinomas, can be described as tumour associated.

*HMFG1, HMFG2:* These two mouse IgG1 antibodies were raised against a delipidated preparation of the human milk fat globule. Both antibodies have a broad spectrum of reactivity with epithelial origin neoplasms, particularly carcinomas

© The Macmillan Press Ltd., 1986

of breast, ovary, bronchus, cervix and gastrointestinal tract (Arklie et al., 1981).

*AUA1:* This mouse IgG1 is directed against an epithelial surface antigen found on some normal epithelia, for example in the colon as well as a wide range of carcinomas including those of colon, breast, bronchus and ovary (Epenetos *et al.*, 1982*b*).

*Antibody guided studies*

These were conducted in two parts. In part 1, pleural effusions were tapped as near to dryness as possible and 1 mCi of radiolabelled antibody was administered by intracavitary injection and washed in with 500 ml of normal saline in patients with pleural effusions and 20 ml of normal saline in patients with pericardial effusions. Gamma camera scans were taken daily, from immediately after injection up to 7 days afterwards in order to calculate the dose of radiation that could be delivered.

In part 2, a higher dose of radiolabelled antibody was given by the intracavitary route as a therapeutic attempt. Patients were kept in a single room for radio protection until their body radioactivity was below 30 mCi. Prior to antibody studies, patients were given potassium iodide 120 mg per day starting one day before part 1, and continuing for 21 days after part 2.

*Dosimetry*

When using radiolabelled antibodies for the treatment of serous effusions it is important to have data on the delivered radiation both to tumour and normal organs. For effective dosimetry it is essential to know the activity at the site, the time course of the activity, the volume of target and the type of radiation emitted by the radionuclide. These data can then be incorporated into a standard formula (Snyder *et al.*, 1978) to produce an answer that although of limited accuracy would be sufficiently useful to judge if the therapeutic procedure were worthwhile or not.

To obtain this a conventional gamma camera was used after calibration with relatively simple phantoms and a small range of calibration factors (Myers *et al.*, 1981). Also a rectilinear scanner with low sensitivity high resolution collimators to image the whole body had been used in the first few days after therapy. The difficulties and limitations of this approach stem from the poor spatial resolution of the camera and the inability to measure accurately the tumour volume. For example, activities distributed in volumes of 1 $mm^3$ or 1 $cm^3$ would appear as having the same area in the image. This degree of inaccuracy would have a drastic effect on dosimetry since knowledge of the *activity per gram* is essential. Therefore, the doses quoted in Table I although they are relatively accurate for normal organs are to be considered inaccurate with regard to tumour doses, the error being in the range of $\pm 50\%$. Accurate tumour doses from antibody-guided irradiation can only be established when dosimetry at the cellular level becomes feasible.

*Immunocytochemistry*

Smears of cells from serous effusions were examined in an indirect immunoperoxidase reaction for antibody reactivity. The antibody/antibodies with highest reactivity was/were selected for use (Epenetos *et al.*, 1982*a*). Immunocytochemistry was considered to be positive when $>50\%$ of neoplastic cells stained with at least one antibody. This was the case with all patients except one (JO).

*Radiolabelling*

Iodine-131 was added to antibody and iodination was carried out in iodogen tubes (Fraker & Speck, 1978). The labelled antibody was separated from free iodine-131 using gel filtration (Sephadex G-50). Specific activity was in the range of 4–8 mCi $mg^{-1}$. There was no detectable loss of antibody immunoreactivity after iodination as tested by ELISA and direct radioimmunoassay, including competition with unlabelled antibody. Samples of radiolabelled antibody were tested for antibody aggregation by gel filtration. There was no evidence of aggregate formation. Prior to patient administration radiolabelled material was millipore filtered and diluted in 1% human serum.

### Results

Ten out of 13 effusions (7 pleural and 3 pericardial) responded favourably to antibody treatment with no reaccumulation of fluid (except for minimal residual fluid), followed up to between 3 and 18 months (mean 7 months) after treatment (Table I). Repeated examination of fluid was performed on 2 patients who gave prior written informed consent. Immunocytology was positive for antibodies HMFG1 and AUA1 before treatment and negative after treatment.

All 3 patients with malignant pericardial effusions responded completely to antibody therapy, with no fluid re-accumulation after 3, 12 and 18 months respectively. Three patients failed to respond to antibody therapy. One patient had an effusion secondary to adenocarcinoma of lung, and he was treated with 46 mCi of iodine-131 labelled

AUA1 antibody. Another patient had a large and recurrent effusion secondary to carcinoma of prostate. He was treated empirically with 21 mCi of iodine-131 labelled HMFG2 antibody without prior immunocytochemical analysis (there were insufficient malignant cells in the fluid for immunocytochemistry). It was interesting that when his recurrent effusion was examined immunocytochemically, malignant cells were negative for the presence of HMFG2 antigen. It is now known that the majority of prostate carcinomas do not express HMFG2 antigen. One patient had a bilateral pleural effusion, secondary to an ovarian carcinoma. Left pleural effusion was treated with 30 mCi of a mixture of HMFG2 and AUA1 antibody, and it recurred two months after treatment. Right pleural effusion was treated with 56 mCi of iodine-131 labelled HMFG2 antibody and has not recurred (follow up 6 months). Nine patients were treated with HMFG2 labelled antibody, 2 with AUA1, 1 with a combination of HMFG2 and AUA1 and 1 with HMFG1, HMFG2 and AUA1 monoclonal antibodies. Three out of 3 pericardial effusions and 7 out of 10 pleural effusions responded completely.

The doses for thyroid ranged from 0.05 to 30 Gy; the explanation for this is that some patients did not have thyroid blockade with potassium iodide and some patients despite taking potassium iodide had some thyroid uptake of free iodine-131. For effective thyroid blockade, we now recommend potassium iodide 120 mg per day starting 3 days before the procedure and taking it for a month. Also potassium perchlorate 400 mg should be taken as a single dose at the time of the procedure.

### Discussion

One of the complications of advanced malignant disease is fluid accumulation in serous cavities. The rate of production varies but in most instances it steadily increases requiring shorter and shorter intervals in the removal of fluid. The intracavitary instillation of colloidal suspensions of radioisotopes is an attempt to control the problem and this approach has been widely used in the past (for review see Hilton *et al.*, 1957) with good results obtained in nearly half the cases.

We have previously shown that the intravenous route of antibody administration leads only to a very small uptake by tumour cells (average $10^{-3}$% of the injected amount $g^{-1}$ tumour) (Epenetos, 1983) and this is the reason why we explored other routes, for example intracavitary administration (Epenetos *et al.*, 1984).

It was our opinion that the better response observed with pericardial effusions was due to the higher dose delivered to pericardium and malignant cells as compared to doses to pleural cavities and malignant cells. It was of interest that in the one patient with bilateral pleural effusions (PB) the one effusion treated with 30 mCi of activity recurred whilst the other effusion treated with 56 mCi of activity did not recur. Nevertheless in other patients (EC, LC, JA) there was a satisfactory remission achieved with doses of $\sim$30 mCi. Therefore, two contributing factors for the outcome of antibody guided-irradiation appear to be (a) the tumour bulk and (b) the delivered dose of iodine-131. From our data, it is suggested that for effective palliation, 30 mCi of iodine-131 labelled antibody should be given intrapericardially and 60 mCi intrapleurally.

This study demonstrates that this new method of treatment of serous effusions is both relatively non-toxic and effective in relieving fluid accumulation and improving the quality of life in patients with advanced malignant disease. At least in one patient (LC) there was prolongation of survival with the patient being alive 2 years after antibody treatment of his pericardial and pleural effusions. It is well known that non-specific agents such as colloidal radioactive phosphorus or other substances such as bleomycin or tetracycline can sometimes be of value in controlling fluid accumulation. The efficacy of this method (10 out of 13 responses) appears, however, superior to previously reported methods (Paladine *et al.*, 1976; Wallach *et al.*, 1975; Strober *et al.*, 1973). Nevertheless, our data at present are not conclusive with regard to the superiority of this method over previous techniques. What is clear, however, is that no patient experienced any toxicity attributable to this therapy. The fact that in one of the three cases where there was fluid reaccumulation the malignant cells were either negative or only weakly positive against the administered antibody, indicates that this new method of treatment may act via specific antigen antibody interaction. Also it highlights potential problems that may arise from antibody therapy such as antigen modulation or emergence of new phenotypes of malignant cells. A randomised study is required to establish conclusively the efficacy and the mode of action of this novel therapeutic approach.

We are grateful to: J. Burchell, A. Cross, K.E. Halnan, J. Lambert, J.P. Lavender, C.J. McKenzie, M. Myers, J.S. Orr, G. Rustin & A. Stewart Ross.

**Table I** Clinical and immunoradiochemical data

| *Patients initials and age* (y) | *Histology* | *Site of effusion* | *Administered dose of 131-I labelled antibody* | *Estimated dose to tumour* (*Gy*) | *Estimated dose to normal organs* (*Gy*) | | *Toxicity* | *Response and follow up* | *Comments* |
|---|---|---|---|---|---|---|---|---|---|
| LB 71 | Ca breast | (L) pleural | 75.0 mCi HMFG2 | 110 | liver | 1.00 | none | CR: 12+ months | — |
| | | | | | spleen | 1.00 | | | |
| | | | | | whole body | 1.00 | | | |
| | | | | | thyroid | 30.0 | | | |
| | | | | | kidney | 0.60 | | | |
| | | | | | heart | 1.00 | | | |
| JA 41 | Ca ovary | (L) pleural | 30 mCi HMFG2 | 50 | liver | 0.60 | slight pyrexia and pain at the site of injection. | CR: 3+ months | 1 month before this treatment the patient's ascites was treated with 100 mCi of labelled HMFG2 and a CR was achieved. |
| | | | | | spleen | 0.60 | | | |
| | | | | | lung | 0.60 | | | |
| | | | | | whole body | 0.40 | | | |
| | | | | | thyroid | 20.0 | | | |
| | | | | | kidney | 0.60 | | | |
| | | | | | heart | 1.00 | | | |
| ER 67 | Ca lung adeno-carcinoma | (L) pleural | 46 mCi AUA1 | 100 | liver | 0.60 | none | failed: 3 months | Died 3 months after therapy from generalised disease. |
| | | | | | spleen | 0.60 | | | |
| | | | | | whole body | 1.00 | | | |
| | | | | | thyroid | 0.05 | | | |
| | | | | | kidney | 0.60 | | | |
| | | | | | heart | 1.00 | | | |
| PB 41 | Ca ovary | (L) pleural | 30 mCi HMFG2 AUA1 | 50 | liver | 1.00 | none | failed: 3 months | — |
| | | | | | spleen | 0.75 | | | |
| | | (R) pleural | 56 mCi HMFG2 | 95 | lungs | 1.00 | | CR: 5+ months | |
| | | | | | whole body | 1.00 | | | |
| | | | | | thyroid | 1.00 | | | |
| | | | | | kidney | 0.75 | | | |
| | | | | | pancreas | 0.80 | | | |
| | | | | | heart | 1.00 | | | |
| AF 39 | Unknown origin: Adeno Ca of lung? | pericardial | 20 mCi HMFG2 | 80 | heart | 2.00 | none | CR: 12+ months | — |
| | | | | | lung | 0.40 | | | |
| | | | | | whole body | 0.40 | | | |
| | | | | | thyroid | 0.05 | | | |
| | | | | | kidney | 0.25 | | | |
| | | | | | liver | 0.20 | | | |
| | | | | | spleen | 0.20 | | | |

| | | | | | | | | | |
|---|---|---|---|---|---|---|---|---|---|
| PP 30 | Ca ovary | (R) pleural | 50 mCi HMFG2 | 100 | liver | 1.00 | none | CR: 3+ months | — |
| | | | | | spleen | 1.00 | | | |
| | | | | | lung | 0.85 | | | |
| | | | | | whole body | 0.70 | | | |
| | | | | | kidney | 0.60 | | | |
| | | | | | thyroid | 30.0 | | | |
| | | | | | heart | 1.00 | | | |
| EC 73 | Ca breast | (R) pleural | 30 mCi HMFG2 | 50 | liver | 0.60 | none | CR: 13+ months | — |
| | | | | | spleen | 0.60 | | | |
| | | | | | lung | 0.60 | | | |
| | | | | | whole body | 0.45 | | | |
| | | | | | thyroid | 0.30 | | | |
| | | | | | heart | 0.60 | | | |
| LC 62 | Ca lung squamous | (L) pleural<br>pericardial | 34 mCi AUA1<br>22 mCi HMFG2 | 40 | liver | 0.60 | none<br>none | CR: 15+ months<br>CR: 16+ months | — |
| | | | | | spleen | 0.60 | | | |
| | | | | | lung | 0.60 | | | |
| | | | | | whole body | 0.50 | | | |
| | | | | | thyroid | 6.00 | | | |
| | | | | | kidney | 0.30 | | | |
| | | | | | pancreas | 0.35 | | | |
| | | | | | heart | 2.00 | | | |
| JA 49 | Ca lung | pericardial | 30 mCi HMFG2 | 40 | liver | 0.60 | none | CR: 2+ months | — |
| | | | | | spleen | 0.60 | | | |
| | | | | | lung | 0.20 | | | |
| | | | | | whole body | 0.30 | | | |
| | | | | | thyroid | 4.00 | | | |
| | | | | | heart | 2.00 | | | |
| PC 48 | Ca pancreas | (R) pleural | 43.9 mCi HMFG1<br>HMFG2<br>AUA1 | 35 | liver | 0.60 | none | CR: 4+ months | Died from generalised disease. There was no reaccumulation of fluid. |
| | | | | | spleen | 0.60 | | | |
| | | | | | lung | 0.60 | | | |
| | | | | | thyroid | 20.0 | | | |
| | | | | | whole body | 0.40 | | | |
| | | | | | kidney | 0.30 | | | |
| | | | | | heart | 0.60 | | | |
| JO 65 | Ca prostate | (R) pleural | 21.49 mCi HMFG2 | 30 | liver | 0.50 | none | failed: 2 months | Died from generalised disease. There was recurrence of pleural effusion. |
| | | | | | spleen | 0.50 | | | |
| | | | | | lung | 0.45 | | | |
| | | | | | thyroid | 5.50 | | | |
| | | | | | whole body | 0.40 | | | |
| | | | | | kidney | 0.30 | | | |
| | | | | | heart | 0.60 | | | |

CR = complete remission.

### References

ANDERSON, C., PHILPOTT, G. & FERGUSON, T. (1974). The treatment of malignant pleural effusions. *Cancer*, **33**, 916.

ARKLIE, J., TAYLOR-PAPADIMITRIOU, J., BODMER, W.F., EGAN, M. & MILLIS, R. (1981). Differentiation antigens expressed by epithelial cells in the lactating breast are also detectable in breast cancers. *Int. J. Cancer*, **28**, 23.

EPENETOS, A.A. (1983). Monoclonal antibodies for the localisation of human neoplasms *in vitro* and *in vivo*. *Proceedings of the First International Symposium on Neutron Capture Therapy*. p. 184.

EPENETOS, A.A., CANTI, G., TAYLOR-PAPADIMITRIOU, J., CURLING, M. & BODMER, W.F. (1982*a*). Use of two epithelium-specific monoclonal antibodies for diagnosis and malignancy in serous effusions. *Lancet*, **ii**, 1004.

EPENETOS, A.A., COURTENAY-LUCK, N., HALNAN, K.E. *et al.* (1984). Hammersmith Oncology Group and the Imperial Cancer Research Fund: Antibody guided irradiation of malignant lesions: three cases illustrating a new method of treatment. *Lancet*, **i**, 1441.

EPENETOS, A.A., NIMMON, C.C., ARKLIE, J. *et al.* (1982*b*). Detection of human cancer in an animal model using radiolabelled tumour-associated monoclonal antibodies. *Br. J. Cancer*, **49**, 1.

FRAKER, P.J. & SPECK, J.C. (1978). Protein and cell membrane iodination with a sparingly soluble chloramide, 1,3,4,6-tetrachloro-5, 6-diphenyl glycouril. *Biochem. Biophys. Res. Commun.*, **80**, 849.

HILTON, G., HALNAN, K.E., HOWARD, N. & GODFREY, B.E. (1957). Effects of the injection of colloidal $^{198}$gold in malignant effusions. *J. Fac. Radiol.*, **8**, 339

MYERS, M.J., LAVENDER, J.P., DE OLIVEIRA, J.B. & MASERI, A. (1981). A simplified method of quantitating organ uptake using a gamma camera. *Br. J. Radiol.*, **54**, 1062.

OSTROWSKI, M. & HALSALL, G. (1982). Intracavitary bleomycin in the management of malignant effusions: A multicentre study. *Cancer Treatement Rep.*, **66**, 1903.

PALADINE, W., CUNNINGHAM, T., SPONZO, R., DONAVAN, M., OLSON, K. & HORTON, J. (1976). Intra cavitary bleomycin in the management of malignant effusions. *Cancer*, **38**, 1903.

SNYDER, W.S., FORD, M.R. & WARNER, G.G. (1978). Estimates of specific absorbed fractions for photon sources uniformly distributed in various organs of a heterogeneous phantom. Society of Nuclear Medicine, New York, p. 5.

STROBER, S., KLOTY, E., KUPERMAN, A. & GOSSEIN, N. (1973). Malignant pleural disease. A radiotherapeutic approach to the problem. *JAMA*, **226**, 296.

WALLACH, H.W. (1975). Intrapleural tetracycline for malignant pleural effusions. *Chest*, **68**, 50.